# Transgenic Mice Over-Expressing ET-1 in the Endothelial Cells Develop Systemic Hypertension with Altered Vascular Reactivity

**DOI:** 10.1371/journal.pone.0026994

**Published:** 2011-11-11

**Authors:** Justin Wai-Chung Leung, Wing Tak Wong, Hon Wai Koon, Fong Ming Mo, Sidney Tam, Yu Huang, Paul M. Vanhoutte, Stephen Sum Man Chung, Sookja Kim Chung

**Affiliations:** 1 Department of Anatomy, The University of Hong Kong, Hong Kong SAR, China; 2 Department of Physiology, Institute of Vascular Medicine and Li Ka Shing Institute of Health Sciences, Chinese University of Hong Kong, Hong Kong SAR, China; 3 Department of Clinical Biochemistry, The University of Hong Kong, Hong Kong SAR, China; 4 Department of Pharmacology and Pharmacy, The University of Hong Kong, Hong Kong SAR, China; 5 Division of Science and Technology, United International College, Zhuhai, China; 6 Research Center of Heart, Brain, Hormone and Healthy Aging, The University of Hong Kong, Hong Kong SAR, China; University of Illinois at Chicago, United States of America

## Abstract

Endothelin-1 (ET-1) is a potent vasoconstrictor involved in the regulation of vascular tone and implicated in hypertension. However, the role of small blood vessels endothelial ET-1 in hypertension remains unclear. The present study investigated the effect of chronic over-expression of endothelial ET-1 on arterial blood pressure and vascular reactivity using transgenic mice approach. Transgenic mice (TET-1) with endothelial ET-1 over-expression showed increased in ET-1 level in the endothelial cells of small pulmonary blood vessels. Although TET-1 mice appeared normal, they developed mild hypertension which was normalized by the ET_A_ receptor (BQ123) but not by ET_B_ receptor (BQ788) antagonist. Tail-cuff measurements showed a significant elevation of systolic and mean blood pressure in conscious TET-1 mice. The mice also exhibited left ventricular hypertrophy and left axis deviation in electrocardiogram, suggesting an increased peripheral resistance. The ionic concentrations in the urine and serum were normal in 8-week old TET-1 mice, indicating that the systemic hypertension was independent of renal function, although, higher serum urea levels suggested the occurrence of kidney dysfunction. The vascular reactivity of the aorta and the mesenteric artery was altered in the TET-1 mice indicating that chronic endothelial ET-1 up-regulation leads to vascular tone imbalance in both conduit and resistance arteries. These findings provide evidence for the role of spatial expression of ET-1 in the endothelium contributing to mild hypertension was mediated by ET_A_ receptors. The results also suggest that chronic endothelial ET-1 over-expression affects both cardiac and vascular functions, which, at least in part, causes blood pressure elevation.

## Introduction

Endothelin-1 (ET-1) is an endothelium-derived peptide with vasoconstriction and mitogenic properties [1]. It exerts its vasoconstrictor effect by binding to G-protein coupled receptors (ET_A_ or ET_B_) [2], [3]. Intravenous injection of ET-1 leads to a transient vasodilatation followed by a sustained increase in blood pressure [4]. Blocking the maturation of ET-1 by intravenous infusion of phosphoramidon lowers blood pressure in spontaneous hypertensive rats (SHRs) [5]. ET-1 also contributes to hypertension in deoxycorticosterone acetate (DOCA)-salt hypertensive rats and DOCA-salt treated SHRs [6]–[8], and ET receptor antagonists (LU135252, A127722.5 and bosentan) had been shown to lower blood pressure in various experimental hypertensive models [9]–[12]. The arterial blood pressure elevation caused by ET-1 is accompanied by structural and functional changes in small arteries including vascular hypertrophy in different models of hypertension 8,13. These pharmacological analyses suggest that ET-1 plays an important role in the pathogenesis of hypertension. However, it is not clear whether these short-term elevation or reduction of ET-1 achieved by drug administration truly simulate the effect of chronic effect of ET-1 under various pathological conditions. Therefore, it is important to verify these studies using genetic approaches to achieve long-term changes in ET-1. This will also avoid the concern of potential side effects of the drugs.

ET-1 gene knockout mice were developed. Unfortunately, they died soon after birth. Paradoxically, heterozygous ET-1 knockout mice were hypertensive [14]. Transgenic mice that overexpress human ET-1 (hET-1) or mouse ET-1 (mET-1) driven by their respective promoters were normotensive even though their plasma and tissue ET-1 levels were slightly elevated [15], [16]. Both hET-1 and mET-1 transgenic mice developed renal cyst, interstitial fibrosis and glomerulosclerosis. Salt-dependent hypertension was observed in the older mET-1 mice, but it was attributed to the consequence of renal damage [16]. Transgenic mice with tyrosine kinase-2 (*tie-2*) promoter directed expression of human ET-1 in the vascular endothelium also exhibited normal arterial blood pressure despite 7-fold increase in plasma ET-1 level. However, these mice showed marked vascular hypertrophic remodeling accompanied by increased production of reactive oxygen species (ROS) [17]. They also had increased inflammatory responses in the endothelium [18]. The lack of hypertension in these ET-1 overexpressing mice may be due to compensatory increased production of the vasodilator NO, as suggested by the fact that introducing the hET-1 transgene into the eNOS gene knockout mice caused further increase in arterial blood pressure [19]. Nevertheless, the role of ET-1 in the development of hypertension remains unclear.

We developed transgenic mice using the tyrosine kinase-1 (*tie-1)* promoter [20] to drive the expression of mouse ET-1 cDNA specifically in vascular endothelial cells. Unlike other ET-1 overexpressing mice described above, these transgenic (TET-1) mice are hypertensive. In the present study we examined the effect of increased ET-1 on vascular and cardiac functions in these mice.

## Results

### Circulating and Pulmonary ET-1 was Increased in TET-1 Mice

ET-1 peptide levels in the plasma, lung, heart, and kidney of the 8-week-old mice were determined by ELISA assays. Heterozygous mice in both transgenic lines showed a small, but not significant increase in plasma ET-1 levels (data not shown). However, significant increases in the plasma ET-1 peptide levels were found in the homozygous mice (*P<0.05) (Table 1). Therefore, homozygous mice were used in all subsequent experiments. Both lines of TET-1 mice showed significant increases in the ET-1 peptide levels in the lungs (**P<0.01) but not in the hearts or kidneys. Such increases in the plasma and lung ET-1 levels persisted in the 1-year-old TET-1 mice (data not shown).

**Table 1:** Endothelin-1 levels in plasma and various major organs.

	NTg	TET-1
**Plasma (pg/ml)**	4.2±0.35	6.0±0.25*
**Lung (pg/mg)**	8.5±0.35	14.2±0.58**
**Heart (pg/mg)**	0.24±0.01	0.28±0.01
**Kidney (pg/mg)**	0.74±0.05	0.78±0.06

ET-1 peptide levels were measured by ELISA. Data are presented as means ± SEM, N = 5. Statistically significant differences are shown as

*P<0.05,

**P<0.01, versus NTg control group.

**Figure 1:**
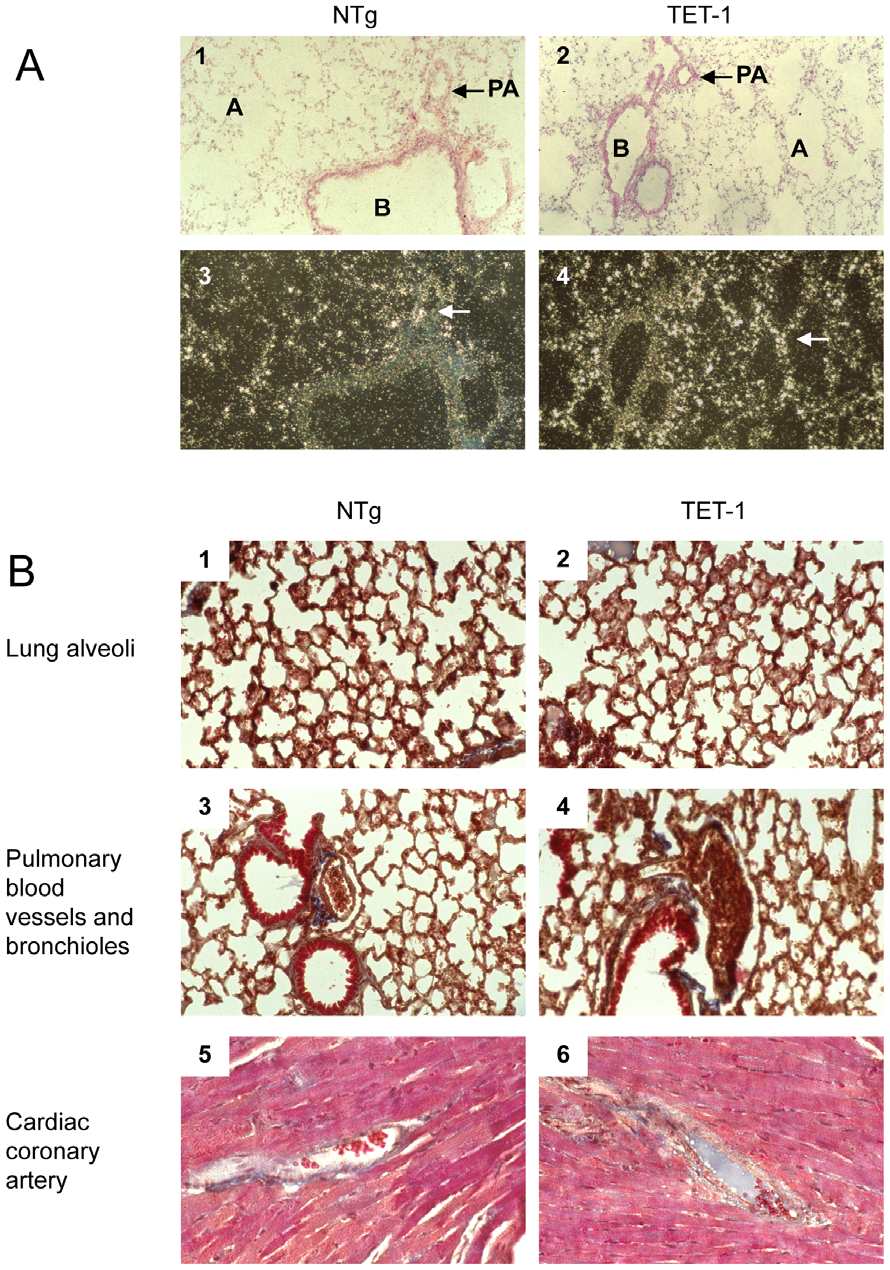
ET-1 transcription and histology in TET-1 lung. (A) Bright-field micrographs of lung from NTg (1), 3771 TET-1 (2) demonstrated the tissue morphologies. In dark field micrographs of lung from NTg (3), 3771 TET-1 (4), ET-1 mRNA expression is represented as white silver grain. A  =  Alveoli; B  =  bronchiole; PA  =  pulmonary arteriole. Arrow in dark field shows ET-1 hybridization signal X 100 magnification. (B) Representative micrographs of tissues stained for collagen deposit (blue) by Masson Trichrome staining including lung alveoli (1–2), pulmonary arterioles and bronchioles (3–4) and heart (5–6). Qualitative observation indicated no obvious change in the sizes and structures of bronchioles, pulmonary arteries among TET-1 and NTg groups (B  =  bronchiole; PA  =  pulmonary arteriole and CA =  cardiac coronary artery) X 100 magnification, N = 5.

To determine the cellular location of ET-1 mRNA expression in the lung, brain and kidney, *in-situ* hybridization was performed using labeled DNA probe which hybridizes to both endogenous and transgene ET-1 mRNA (Fig. 1A). Endothelial specific ET-1 hybridization signal was found only in the small vessels of TET-1 lungs, and in the lung of non-transgenic (NTg) mice. No ET-1 mRNA expression was found in epithelial cells of the bronchial structure, indicating that the ET-1 transgene was expressed specifically in the endothelial cells.

### Over-expression of Endothelial ET-1 Did Not Lead to Fibrosis

In rats, monocrotaline induces pulmonary hypertension, collagen V deposition and perivascular fibrosis, and these were thought to be mediated by ET-1 [21]. To determine if over-expression of ET-1 in the lungs of TET-1 mice would lead to similar pathologies, Masson Trichrome staining was employed to examine sections of their lungs. The lungs of 6 months old TET-1 mice did not exhibit any abnormality in size, shape and structure of the alveoli (Fig. 1B_1–2_). The pulmonary vascular and bronchiolar structures appeared normal (Fig. 1B_3–4_) and there was no sign of perivascular fibrosis or excessive collagen deposition. In addition, the coronary arteries had a similar morphology to those of their NTg counterparts (Fig. 1B_5–6_).

### TET-1 Mice Exhibited Hypertension that was Mediated by ET_A_ Receptors

**Figure 2:**
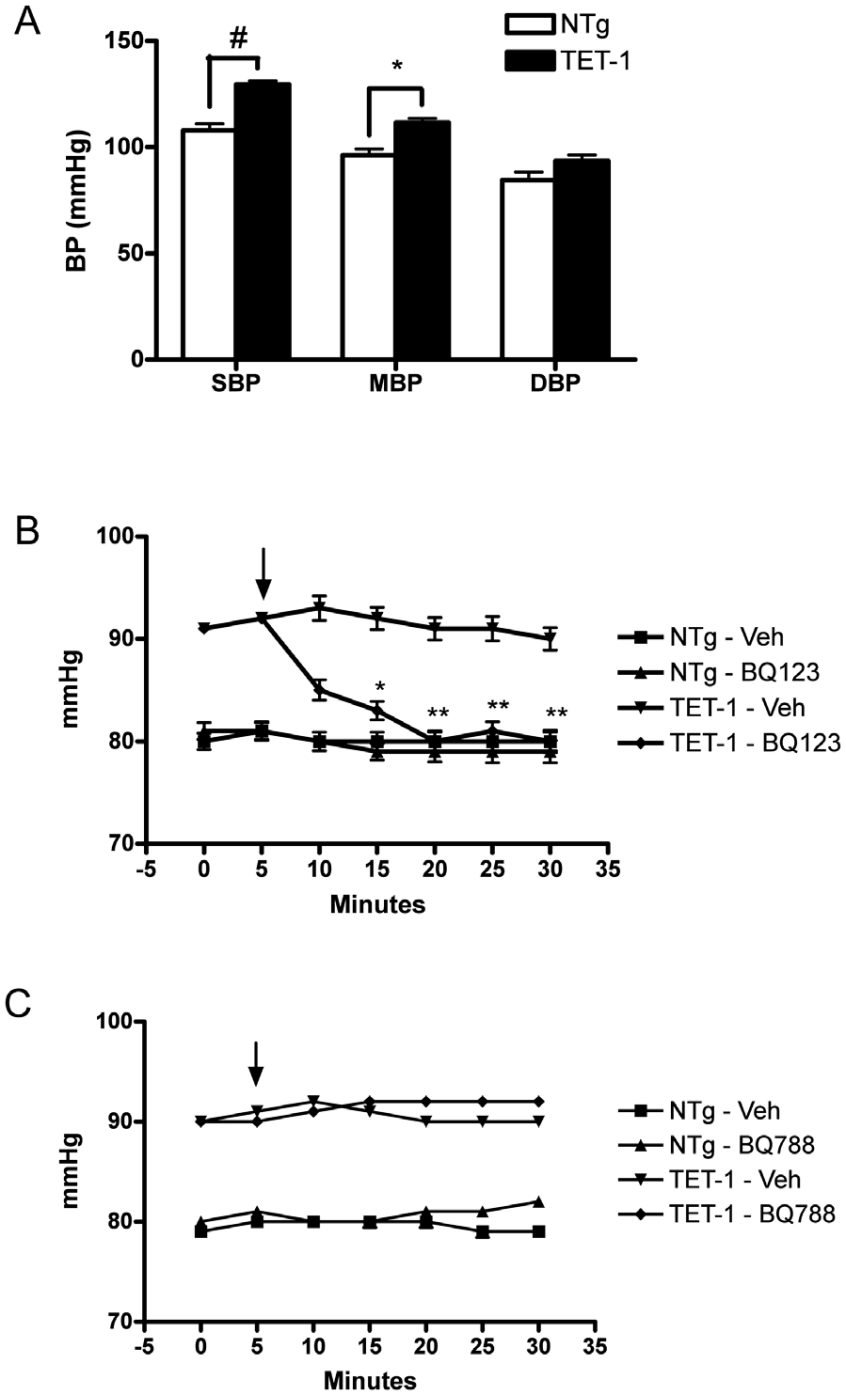
Elevated blood pressure in TET-1 mice. (A) Tail cuff measurement showed systolic blood pressure, mean blood pressure and diastolic blood pressure of 8-week-old TET-1 mice and NTg counterparts (N = 12). (B and C) Mean arterial blood pressure (MABP) was measured with polyethylene catheter in the right common carotid artery under anesthesia. (B) BQ-123 (1 mg/kg) and (C) BQ-788 (1 mg/kg) were injected intraperitoneally at the time indicated by the arrows. The hypertension in TET-1 mice from line 3771 (B) was inhibited by BQ-123 but not by BQ788 (C). Means±SEM (mmHg), *P<0.05, **P<0.01, #P<0.0001 when compared to vehicle control group of the same genotype, N = 3.

To determine whether endothelial cells specific ET-1 over-expression leads to blood pressure elevation, tail cuff measurement was performed. TET-1 mice showed higher SBP and MBP compared to the NTg counterparts. The diastolic blood pressure was not significantly different (Fig. 2A).

We measured mean arterial blood pressure (MABP) of the 8 weeks old TET-1 mice using polyethylene pressure sensing catheter. As shown in Figure 2B and 2C, MABP of the TET-1 mice was significantly higher than that of the NTg mice. To determine the receptor subtype that mediates hypertension in the TET-1 mice, the TET-1 and NTg mice were treated with ET_A_ and ET_B_ receptor antagonists. Administration of ET_A_ receptor antagonist, BQ123 (1 mg/kg body weight) had no effect on the MABP in NTg mice (Fig. 2A). On the other hand, MABP of the TET-1 mice was reduced to the basal level of the NTg mice within 20 minutes of BQ-123 treatment. Administration of ET_B_ receptor antagonist, BQ788 had no effect on the blood pressure of TET-1 and NTg mice (Fig. 2C).

### TET-1 Mice Developed Left Ventricular Hypertrophy

The left ventricle to total heart wet weight ratio in TET-1 mice was 17% higher than that of their NTg littermates (P<0.001, ANOVA) (Table 2). The left ventricle of both lines heterozygous transgenic mice appeared normal (data not shown). For both lines of transgenic TET-1 mice, the right ventricle to total heart wet weight ratio was similar to that of NTg mice.

**Table 2:** Physiological parameters of heart and electrocardiography.

	NTg	TET-1
**Body weight (g)**	22.6±0.51	22.9±0.39
**MABP (mmHg)**	79.1±1.44	94.3±1.91**
**Whole heart (mg)**	128.1±4.81	133.5±3.90
**LV/Hr (mg/mg)**	0.34±0.01	0.40±0.01***
**RV/Hr (mg/mg)**	0.17±0.01	0.16±0.01
**ECG**		
**P-P (ms)**	127.1±3.58	124.9±3.18
**P-R (ms)**	25.6±1.56	33.3±1.16**
**P-R/P-P ratio**	0.21±0.02	0.27±0.01*
**QRS (ms)**	7.0±0.29	7.8±0.25
**Electrical axis (angle)**	52.7±9.1	-14.8±16.0**
	Normal	Left deviation

Body and heart weight (means±SEM) of TET-1 and NTg at 8-weeks old. Mean arterial blood pressure (mmHg, means±SEM) of TET-1 and NTg mice. LV/Hr, ratio of left ventricular wall wet weight to whole heart wet weight. RV/Hr, ratio of left ventricular wall wet weight to whole heart wet weight. Left ventricular walls of TET-1 were heavier than those of non-transgenic mice. Electrocardiographic (ECG) profiles of TET-1 and non-transgenic (NTg) mice. P-P and P-R intervals were calculated by time distance between respective peaks of ECG tracings. QRS intervals were measured from beginning to end of the QRS complex. P-R/P-P is the ratio of P-R interval to P-P interval. Electrical axis with (0°)–(90°) is defined as normal, (−1°)–(−90°) is defined as left axis deviation. TET-1 show increased P-R interval and left axis deviation. Values are expressed in means±SEM, N = 9–12.

*P<0.05,

**P<0.01,

***P<0.001 (ANOVA), compared to NTg littermates. MABP – mean arterial blood pressure, LV – left ventricle, RV – right ventricle, ECG – electrocardiogram.

The electrocardiogram (ECG) showed that both lines of TET-1 mice had a left electrical axis deviation (Table 2). No arrhythmia was observed. The atrial-ventricular conduction was found to be delayed in the TET-1 mice. The P-R intervals of TET-1 mice (33 ± 1.2 ms) were significantly increased compared to their NTg littermates (26 ± 1.6, *P<0.05, **P<0.01, ***P<0.001). The ratio of P-R/P-P interval was significantly increased (P<0.05, ANOVA), indicating delayed arterial-ventricular conduction in the TET-1 mice.

### Kidney Function Analysis

Renal dysfunction is one of the hallmarks of hypertension. To evaluate the renal function, urine and serum samples were collected for solute measurement and clearance study. No significant difference was observed in water and chow consumption between different groups (Data not shown) Urinary concentration of sodium, potassium, calcium and chloride ions as well as urea and creatinine were not statistically different between the NTg and TET-1 groups. The total amount of these urinary solutes excreted in 24 hr was also the same in two groups. However, it is noteworthy that higher levels of NO and urea were found in the sera of TET-1 mice (Table 3).

**Table 3:** Urine and serum solutes of euhydrated NTg and TET-1 mice.

Sample and substance	NTg	TET-1
**Urine**	Concentration	
**Osmolality** *(mmol/kg)*	2817.6±132.06	2690.7±143.85
**Na** *(mmol/L)*	182.9±7.84	158.4±12.87
**K** *(mmol/L)*	468.5±21.32	465.1±21.94
**Ca** *(mmol/L)*	2.71±0.19	3.01±0.17
**Cl** *(mmol/L)*	322.2±15.43	292.6±16.78
**Urea** *(mmol/L)*	1479.8±80.10	1470.5±80.79
**Creatinine** *(µmol/L)*	4716.5±262.99	4593.6±358.26
**Serum**	Concentration	
**Osmolality** *(mmol/kg)*	315.64±1.89	323.3±5.19
**NO** (µM)(nitrate+nitrite)	27.14±1.88	38.86±2.9*
**Na** *(mmol/L)*	144±0.81	144.9±0.60
**K** *(mmol/L)*	3.62±0.11	3.7±0.14
**Ca** *(mmol/L)*	1.792±0.06	1.816±0.05
**Cl** *(mmol/L)*	110.14±0.45	108.7±0.57
**Urea** *(mmol/L)*	6.94±0.20	8.03±0.27**
**Creatinine** *(µmol/L)*	13.57±0.88	15.3±0.74
**Urine**	24 h excretion	
**Volume** *(ml)*	1.07±0.11	1.12±0.88
**Na** *(mmol)*	0.16±0.01	0.14±0.01
**K** *(mmol)*	0.42±0.019	0.41±0.02
**Ca** *(mmol)*	0.0024±0.0002	0.0027±0.0001
**Cl** *(mmol)*	0.29±0.013	0.26±0.01
**Urea** *(mmol)*	1.33±0.072	1.32±0.07
**Creatinine** *(µmol)*	4.24±0.23	4.13±0.32

Urine and serum solutes of TET-1 and NTg mice. Values are means ± SEM (N = 15), *P<0.05,

**P<0.01 compared with NTg mice.

### Endothelium-dependent Vascular Function in TET-1 Mice

**Figure 3:**
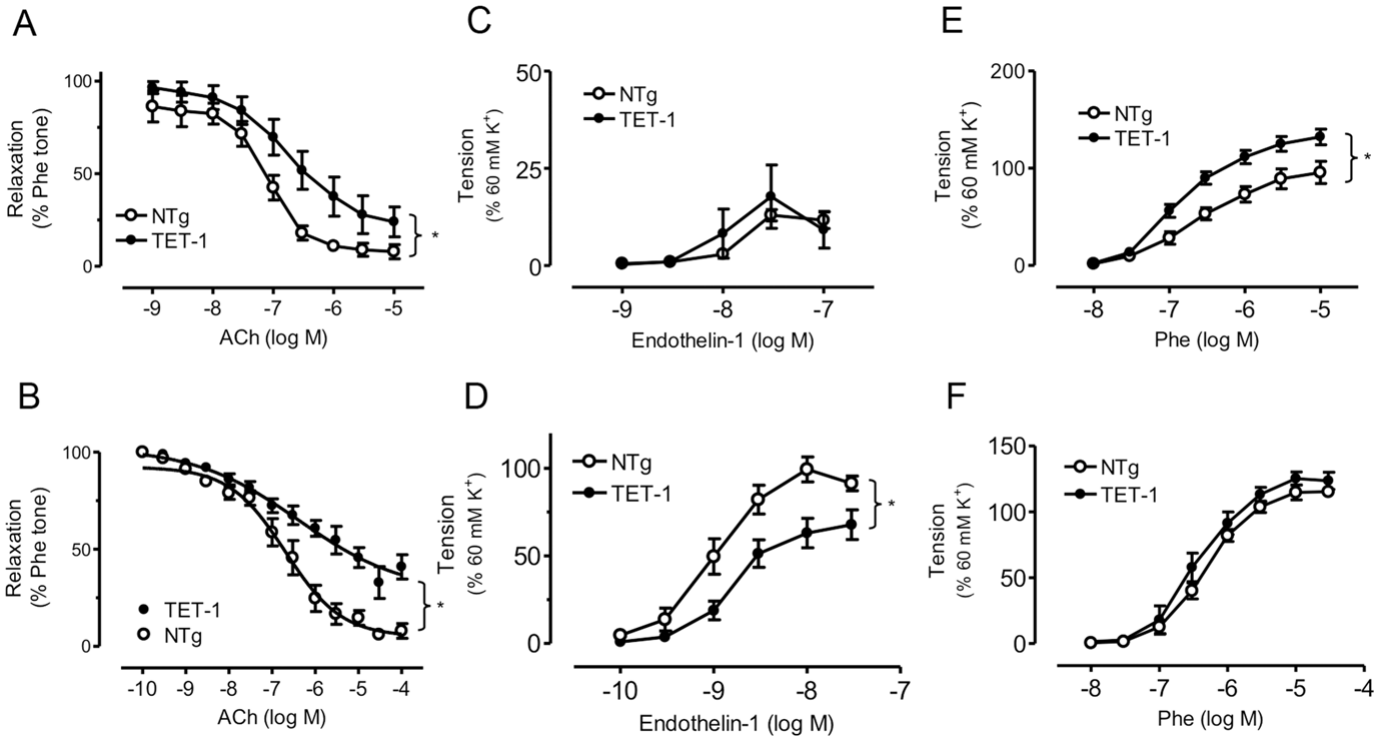
Vascular reactivity in aortas and mesenteric arteries of 8 weeks old TET-1 mice. Cumulative concentration-response curves to (A and D) acetylcholine (ACh), (B and E) ET-1 and (C and F) Phe. Relaxations are percent decrease in tension during contractions to phenylephrine. Contractions are percent reduction in lumen. (A to C) Aortic segments and (D–F) mesenteric artery in response to ACh, ET-1 and Phe in TET-1mice and NTg littermates. Results are Means±SEM, N = 8–10. *P<0.05.

Endothelium-dependent relaxation to acetylcholine was significantly attenuated in both aorta and mesenteric artery of TET-1 mice compared to the NTg mice (Fig. 3A, aorta: AUC, 274.4±25.15 versus 193.5±13.2, P<0.01; Fig. 3B, mesenteric artery: AUC, 91.58±8.03 versus 144.1±17.6, respectively; P<0.05). On the other hand, sodium nitroprusside-induced endothelium-independent relaxations were not significantly different between arteries of TET-1 and NTg mice (data not shown).

ET-1-induced contraction of the mesenteric artery were blunted in the TET-1 mice compared to that of NTg mice (Fig. 3D, mesenteric artery: AUC, 91.58±8.03 versus 144.1±17.6, respectively; P<0.05) There was no significant difference between TET-1 and NTG mice in ET-1 induced contraction of the aorta (Fig. 3C). In the TET-1 aorta, but not the mesenteric artery, phenylephrine-induced contraction was increased significantly (Fig. 3E, aorta: AUC, 233.3±15.41 versus 162.6±17.58, P<0.01). The TET-1 and NTG mice showed no difference in their aorta and mesenteric artery's response to thromboxane agonist U46619 (data not shown).

## Discussion

ET-1 is thought to contribute to increased arterial pressure in several hypertensive animal models [13]. However, this is primarily deduced from experiments with pharmacologically manipulated short-term changes in ET-1 level. We therefore developed transgenic mice that overexpress ET-1 to simulate chronic elevation of ET-1 in some pathological conditions. The *tie-1* promoter was used to drive the expression of mouse preproET-1 specifically in vascular endothelial cells. Two independent lines (3771 and 3796) of TET-1 mice were investigated. Both lines showed higher levels of ET-1 in their sera and lungs. The increased ET-1 levels were still observed in the 1 year-old TET-1 mice indicating that the ET-1 transgene expression was maintained throughout adult live. The increase ET-1 expression in the lungs of the TET-1 mice was confirmed by *in situ* hybridization and ELISA. Despite the purported mitogenic and fibrotic effects of ET-1 [22], we did not find fibrosis in TET-1 mouse lungs or any structural abnormality in various main organs at age of 6 months [23], [24]. Both lines of mice exhibited higher MABP, which was normalized by the administration of an ET_A_ receptor antagonist (BQ123) but not by the ET_B_ antagonist (BQ788), indicating that ET-1-induced hypertension in these mice was mediated by ET_A_ receptors. On the other hand, administration of BQ123 to the NTg mice had no effect on their MABP, suggesting that ET-1 was not involved in basal blood pressure regulation under normal condition. This is in agreement with previous reports which showed that systemic blood pressure in healthy humans was not affected by BQ123 [25].

It is not clear why other ET-1 overexpressing mice previously reported did not have increased MABP whereas TET-1 mice did. The main difference is the promoter used to drive the expression of ET-1. We used the tie-1 promoter which drives the expression of ET-1 in the endothelial cells of small blood vessels only. Transgenic ET-1 driven by the mouse or the human ET-1 promoter is likely to be expressed in wider range of tissues. Even the tie-2 promoter is active in a wider range of cell types than tie-1 promoter. It is known to express transgenes in endothelial cells of the arteries, veins and capillaries in various organs [26]. It is likely that overexpression of ET-1 in some of these cells might induce compensatory increased expression of vasodilator such as NO to counteract the effect of increased level of ET-1. The fact that MABP was increased in the heterozygous general ET-1 gene knockout mice [14], and decreased in vascular endothelial cell-specific ET-1 gene knockout mice [27], supports the notion that certain cell types have feedback mechanisms to compensate for increase/decrease ET-1 level.

The TET-1 mice showed left ventricular hypertrophy and a left shift of the electrical axis in ECG analysis. These are not observed in other ET-1 overexpressing transgenic mice that do not have increased MABP and they are likely to be due to cardiac remodeling as an adaptation to the higher MABP. Hypertension in the TET-1 mice probably caused mild chronic overloading of the heart. Increased dP/dt in TET-1 mice suggests load-dependent increase of heart contractility in response to increased vascular resistance. Increased heart contractility is often associated with increased weight of the left ventricular wall. Left electrical axis deviation seen in TET-1 mice suggests that there was a block in impulse conduction. These mice also exhibited prolonged P-R interval, often associated with essential hypertension and indicative of a delay in depolarization from sinoatrial node to the ventricular muscle [28]. These are indicators of mild heart diseases with no immediate threat to life which explained why TET-1 mice did not show early mortality. Despite higher level of ET-1 in the lungs of TET-1 mice they did not have right ventricular hypertrophy, suggesting no pulmonary hypertension.

All three previous reported ET-1 overexpressing transgenic mouse lines mice developed renal interstitial fibrosis, renal cysts, and glomerulosclerosis, leading to reduced glomerular filtration rate (GRF) [15], [16]. These renal lesions were not observed in the TET-1 mice. However, their serum urea levels were slightly elevated, but creatinine clearance appeared normal. Together, these suggest that the TET-1 mice may have very mild renal dysfunction, but their elevated blood pressure was most likely not of renal origin. The isolated arteries from these mice exhibited blunted endothelium-dependent responses. This vascular dysfunction probably contributed to their hypertension.

It had been shown that chronic subcutaneous infusion of ET-1 increases acetylcholine-induced relaxation in the rat aorta [29]. By contrast, in the TET-1 mice, the endothelium dependent relaxation response in aorta was attenuated whereas the response in smooth muscle was augmented. This dramatically illustrates the different effects of raising plasma ET-1 level by different means. Endothelial release of ET-1 in the TET-1 mice should be a better model than subcutaneous infusion of ET-1 to simulate chronic pathological increase of ET-1. Increased peripheral resistance is a hallmark of essential hypertension attributed mainly to abnormalities in small arteries 30,31. The TET-1 mice, which overexpress ET-1 specifically in the endothelial cells of small arteries, developed hypertension. They should provide a good animal model to investigate the role of ET-1 in the pathogenesis of hypertension.

## Materials and Methods

### Animals

The generation of TET-1 mice was reported earlier [32]. Transgenic animals and their wild-type littermates were kept under controlled conditions at a temperature of 20^o^C and housed under diurnal light condition. They were fed on standard rodent chow and water ad libitum, and unless specified otherwise, were used for experiments at the age of 8 weeks. The use of animals in this study was conducted according to the requirements of the Cap. 340 Animal (Control of Experiments) Ordinance and Regulations, and all relevant legislation and Codes of Practice in Hong Kong. All the experimental and animal handling procedures were approved by the Committee of the Use of Live Animals in Teaching and Research in The University of Hong Kong (Permit number #634-01).

### Quantitative Measurement and Localization of ET-1 Expression in The Lung

In situ hybridization was performed as described [33] on tissues of 8 weeks old TET-1 mice to determine the site of transgene expression. Levels of plasma and tissue ET-1 peptide were assessed by ELISA using an ET-1 enzyme immunoassay kit RPN 228 (Amersham, Buckinghamshire, U.K.) as described. [34].

### General Histology and Masson-Trichrome Staining for Collagen

Heart, lung and kidney tissues were collected from 6-month-old mice. The tissues were embedded in paraffin and the dewaxed slides were first immersed in Weigert's hematoxylin (Sigma, St. Louis, MO, U.S.A.). They were stained serially with acid fuschin, phosphomolybdic acid and methyl blue. The color was fixed in 1% acetic acid. Then the slides were dehydrated using increasingly higher concentration of alcohol, fixed in toluene, mounted in Permount (Fisher Scientific, U.S.A.) and air-dried overnight before observation and photography [35]. The nuclei appear as blue-black while the cytoplasm, muscle fibers and red blood cells were stained red and the collagen fibers stained blue. The stained sections were viewed and photographed with a light microscope (Axiophot, Zeiss, Germany) using bright-field and/or dark-field illumination.

### Systemic Blood Pressure Measurement

Eight weeks old male mice were anesthetized with urethane (1.8 mg/g body weight) and the body temperature was maintained at 37.0 – 38.0°C on a heating pad with automatic temperature control (FHC, Bowdoinham, ME, U.S.A.). The mean arterial blood pressure was monitored continuously by cannulating the right common carotid artery with a polyethylene catheter connected to a saline-filled pressure transducer (Model 1050BP, UFI, Morro Bay, CA, U.S.A.) for one hour. Heart rate and mean arterial blood pressure data were analyzed using the PowerLab 8 s software (ADinstruments, NSW, Australia). To determine the direct hemodynamic effects of the selective ET_A/_ET_B_ receptor antagonist BQ123/BQ788 (Peninsula Co., San Carlos, CA, U.S.A.) on basal mean arterial blood pressure (MABP), the drugs were administered (1 mg/kg) intraperitoneally (100 µl). Control groups were injected with 100 µl saline. After drug administration, MABP was continuously monitored and recorded for 30 minutes.

### Tail Cuff Blood Pressure Measurement

Blood pressure was determined in conscious animals using a non-invasive computerized automated tail-cuff system (Vistech BP-2000 Blood Pressure Analysis System; Vistech Systems; Apex, NC). The mice were trained by placing them in restrainers for 15 minutes daily for five consecutive days before determining their blood pressure. On the day of measurement, the animals were placed on a heated platform and underwent 10 preliminary cycles. The average of three 10-cycle measurements, which each have a minimum of 6 out of 10 successful measurement cycles, was used for data analysis. BP-2000 software calculated the systolic (SBP) and diastolic (DBP) blood pressure.

### Electrocardiogram (ECG)

Electrocardiographic recordings were acquired from a single channel ECG recorder (Cardisuny 501B, Fukuda M-E, Japan) with the filter, sensitivity set at 2 cm/mV, and at 50 mm/s speed. The mice were anesthetized using Hypnorm & Hyponorval mixture (fentanyl citrate 0.394 mg/kg, fluanisone 12.5 mg/kg and midazolam 6.25 mg/kg) and were placed on a heating plate to maintain body temperature around 37.0–38.0°C. One electrode was placed at the base of each limb using subcutaneous platinum pins. Signals was amplified and recorded by a PowerLab 8s system (ADinstruments, New South Wales, Australia). Recordings were made for 10 seconds and twice, each for leads I, II, III, aVR, aVL and aVF. The P-P and P-R time intervals were determined by measuring the distances between respective peaks of the ECG waves. Three consecutive beat intervals in lead I were sampled and averaged. Heart rate was derived from the P-P interval. Only data with the heart rate between 400-600 beats per min were accepted [36]. The electrical axis was determined by two-dimensional vector ECG. The algebraic sums of voltage amplitudes of the QRS complex in lead I and III were used to calculate the degree of shift of the electrical axis.

### Heart Wet Weight

Hearts were removed, and washed in cold PBS to remove excess blood. The major blood vessels and the adhering fat were removed. The whole hearts were blotted and weighed. The ventricles were cut free from the atria. The left and right ventricles were separated from the septum. Then the atria, ventricles and septum were blotted again, and their wet mass was recorded to the nearest 0.1 mg on an electronic balance [37].

### Evaluation of Renal Function

Individual mice were put into a metabolic cage to measure water/chow consumption, urine output and to collect urine sample (Nalgene, U.S.A.). Serum samples were collected by cardiac puncture under anesthesia (ketamine/xylazine). Serum Na, K, Ca, Cl, urea and creatinine were measured by means of a Hitachi-747 Autoanalyzer (Boehringer-Mannheim, Mannheim, Germany). Serum nitric oxide level was measured with Griess reagent assay (Cayman Chemical, Ann Arbor, MI, U.S.A.). Urine Na, K, Ca, Cl, urea and creatinine were measured with a Synchron CX5 Analyzer (Beckman Instruments, Inc., Fullerton, CA, USA). Urine osmolality was measured by the vapor pressure method using a Wescor Vapro vapor pressure osmometer (Wescor Inc., Logan, Utah, USA). The creatinine clearance was used as an index of glomerular filtration rate and was calculated from the formula: creatinine clearance = urinary creatinine x urine volume/serum creatinine.

### Functional Studies on Isolated Arteries

Mice were anesthetized with ketamine/xylazine. The abdominal aortae and the second branch mesenteric arteries with adjacent tissue were collected and placed in ice-cold physiological salt solution (Krebs solution of the following composition (mM): NaCl 119, KCl 4.7, CaCl_2_ 2.5, MgCl_2_ 1, KH_2_PO_4_ 1.2, NaHCO_3_ 25 and glucose 11). The adhering fat was removed and the arteries were cut into segments of 2 mm in length. The rings were suspended between two tungsten wires with a diameter of 40 µm for aortae and 25 µm for mesenteric arteries in an organ chamber filled with control solution and oxygenated with 95%O_2_ and 5% CO_2_ to give a pH value between 7.3–7.5. The organ chamber was maintained at 37°C using a built-in heat exchanger. One of the tungsten wires was connected to a movable holder supporting a tension transducer so that isometric force measurement could be collected by a data acquisition system (PowerLab 4SP, ADInstruments, U.S.A.). An optimal basal tension of 3mN for aortae and 1 mN for mesenteric arteries was applied and the arteries were allowed to equilibrate at this baseline tone for 90 minutes. After stabilization, the segments were exposed to KCl (60 mmol/L) twice for contraction control. Endothelium-dependent and -independent relaxations were assessed by measuring decrease in tension caused by cumulative doses of acetylcholine (ACh, 1 nmol/L to 10 µmol/L) and sodium nitroprusside (SNP, 10 nmol/L to 0.1 µmol/L) during contraction to phenylephrine (3 µmol/L). Contractions were evoked with ET-1 (0.1 nmol/L to 0.1 µmol/L) and phenylephrine (10 nmol/L to 10 µmol/L).

### Statistical Analysis

Data are presented as means ± standard error of mean (SEM) with N being the number of individual observations. Statistical analysis was performed using Prism 4.0 software (Graphpad, San Diego, CA, U.S.A.) and Statview 5.0 software (SAS Institute, Cary, NC, U.S.A.). Numerical data sets from all experiments were analyzed using the Mann-Whitney U-test for two groups comparison and One-way ANOVA for multi-groups comparison, as appropriate. The area under each dose-response curves was estimated and statistical significance between the areas under curves of different datasets was analysed using Student's t-test for comparison of two groups. P values less than 0.05 was considered to indicate statistically significant difference.
